# Podocyte Injury in Lupus Nephritis

**DOI:** 10.3390/jcm8091340

**Published:** 2019-08-29

**Authors:** Hamza Sakhi, Anissa Moktefi, Khedidja Bouachi, Vincent Audard, Carole Hénique, Philippe Remy, Mario Ollero, Khalil El Karoui

**Affiliations:** 1 AP-HP (Assistance Publique des Hôpitaux de Paris), Department of Nephrology and Renal Transplantation, Groupe Hospitalier Henri-Mondor, 94010 Créteil, France; 2 UPEC (Université Paris Est Créteil), UMR-S955, 94010 Créteil, France; 3 INSERM (Institut National de la Santé et de la Recherche Médicale) U955, Institut Mondor de Recherche Biomédicale (IMRB), Équipe 21, 94010 Créteil, France; 4 AP-HP (Assistance Publique des Hôpitaux de Paris), Department of Pathology, Groupe Hospitalier Henri-Mondor, 94010 Créteil, France

**Keywords:** lupus nephritis, lupus podocytopathy, podocytes, nephrotic syndrome, crescent

## Abstract

Systemic lupus erythematosus (SLE) is characterized by a broad spectrum of renal lesions. In lupus glomerulonephritis, histological classifications are based on immune-complex (IC) deposits and hypercellularity lesions (mesangial and/or endocapillary) in the glomeruli. However, there is compelling evidence to suggest that glomerular epithelial cells, and podocytes in particular, are also involved in glomerular injury in patients with SLE. Podocytes now appear to be not only subject to collateral damage due to glomerular capillary lesions secondary to IC and inflammatory processes, but they are also a potential direct target in lupus nephritis. Improvements in our understanding of podocyte injury could improve the classification of lupus glomerulonephritis. Indeed, podocyte injury may be prominent in two major presentations: lupus podocytopathy and glomerular crescent formation, in which glomerular parietal epithelial cells play also a key role. We review here the contribution of podocyte impairment to different presentations of lupus nephritis, focusing on the podocyte signaling pathways involved in these lesions.

## 1. Introduction

Systemic lupus erythematosus (SLE) is a chronic immune complex-mediated disease characterized by disseminated inflammation that may affect multiple organs. Renal lesions occur in 30 to 70% of patients with SLE and have a large impact on disease prognosis, resulting in a risk of end-stage renal disease (ESRD) of 10% after five years of follow-up [1,2]. Lupus nephritis (LN) is associated with a wide spectrum of kidney lesions, mostly characterized by glomerular involvement.

The International Society of Nephrology/Renal Pathology Society (ISN/RPS) developed a classification system in 2004 to increase reproducibility and to guide therapeutic management according to the underlying glomerular lesions [3]. The classification of LN is based principally on the presence of glomerular proliferative lesions (mesangial, endocapillary and extracapillary proliferation) and the localization of immune-complex (IC) deposits (Table 1). 

LN results principally from IC deposition accompanied by complement activation, which triggers tissue inflammation, leading to glomerular injury. Abnormal adaptive and innate immune responses induce the release of inflammatory mediators, such as interferon-α, which amplify glomerular lesions [5,6]. These processes take place in glomeruli and may account for the various clinical, biological, and histological features of renal disease. Many recent studies have highlighted the importance of podocyte (visceral epithelial cell) injury in lupus glomerulonephritis [4,7] (Table 1).

The glomerular capillary wall includes fenestrated endothelial cells, the glomerular basement membrane (GBM) and podocytes, on the side of Bowman’s space. Podocytes are highly differentiated epithelial cells anchored to the basement membrane through foot process extensions, which interact with those of adjacent podocytes to form the slit diaphragm, the ultimate filtration barrier. This slit diaphragm is a unique cellular junction formed from podocyte-specific proteins, such as nephrin and podocin, which interact with the actin cytoskeleton [8]. Indeed, the actin cytoskeleton is a major structure in the podocyte contributing to the formation and dynamics of foot processes [9]. Molecular events disturbing the integrity of the actin cytoskeleton can lead to foot process effacement (FPE) with secondary podocyte detachment, a key event associated with the occurrence of heavy proteinuria [10]. FPE may reflect reorganization of the actin cytoskeleton, which may be seen as a protective mechanism preventing podocyte detachment following various types of injury [11]. FPE is a common finding in idiopathic nephrotic syndrome with minimal change disease (MCD) or primary focal and segmental glomerulosclerosis (FSGS), but it may also occur in many other glomerulopathies [8,10].

We review here the involvement of podocyte injury in the different classes of LN, highlighting the key role of these cells regardless of the initial immune trigger of disease.

## 2. Podocyte Injury in Immune-Complex Lupus Nephritis

The localization of IC deposits and their ability to trigger inflammation underlie the mechanisms of glomerular injury in LN [12]. Deposits of immunoglobulins and complement appear in mesangial structures and may be localized to subendothelial and/or subepithelial compartments. Subendothelial deposits are more likely to trigger inflammatory reactions than subepithelial deposits on the outside of the GBM. Anti-DNA antibodies induce complement activation and inflammatory reactions [5], and they are associated with the most severe classes of LN (class III and class IV) [3]. Membranous LN (class V) is characterized by subepithelial deposits, leading to local complement activation and podocyte dysfunction, but typically with little or no inflammatory reaction [13]. 

There is compelling evidence to suggest that podocytes are a direct or indirect target of IC deposits in LN. Histological podocyte injury is observed in various LN classes [14]. FPE is associated with severe proteinuria, in both non-proliferative and proliferative forms [14,15]. The expression of mature podocyte markers, such as synaptopodin, nephrin, and glomerular epithelial protein 1 (GLEPP1), is lost in proliferative forms of LN [16]. Similarly, a downregulation of podocyte marker expression has been reported at early stages of disease in a murine model of LN [17], suggesting a possible role for podocyte dysfunction in the development of histological lesions. This modulation of podocyte marker expression is observed at both the protein and mRNA levels, and urinary sediment analyses in patients with active LN have shown that the levels of nephrin, podocin, and synaptopodin mRNA are correlated with lupus activity [18,19] 

Lupus auto-antibodies are known to recognize several antigens on the mesangium and glomerular capillary wall. Chromatin (released from apoptotic intraglomerular cells) bound to the GBM was initially identified as a target of anti-DNA antibodies [20,21]. It was subsequently shown in an experimental model of LN that only anti-dsDNA antibodies bound to GBM activated complement and induced proteinuria, with no such effect observed with non-GBM-binding anti-DNA antibodies [22]. This suggests that the direct binding of anti-DNA antibodies to specific antigens in the GBM or mesangial matrix might be pathogenic [22]. Mesangial cells and podocytes have also been identified as direct targets of IC through specific epitopes (notably annexin II) [23]. Moreover, several podocyte antigens have been identified as potential autoantibody targets in LN. For example, alpha-actinin 4 was first identified as a potential target in a lupus-prone model [24]. Antibodies raised against alpha-actinin 4 have also been purified from the sera of LN patients but not from non-LN patients [25]. Proteomic studies of renal biopsy samples from LN patients recently led to the identification of glomerular IgG recognizing podocyte antigens, such as alpha-enolase and annexin A1 [26].

Lupus-associated podocyte injury may occur via several mechanisms, including podocyte dedifferentiation and cell death. It has been suggested that lupus podocytopathy involves cell detachment mechanisms, based on the finding that podocyturia is correlated with LN activity [27]. The cause of podocyte detachment remains a matter of debate, but it may involve in situ apoptosis or a loss of anchoring to the GBM [8]. However, it has been reported that 60% of urinary podocytes are viable, and this observation is more consistent with an active detachment process than an apoptotic mechanism [19]. Focal adhesions contribute to podocyte attachment and signal transduction through their main component, α3β1 integrin, a transmembrane protein involved in podocyte anchoring to the GBM [9]. Exposure to TGF–β in vitro induces a decrease in podocyte adhesion and an increase in apoptosis, due to the downregulation of α3β1 integrin [28]. “Mitotic catastrophe”, which is characterized by binucleate podocytes and cell detachment, may also underlie podocyte detachment in this context [8,29]. It has been suggested that podocytes cannot proliferate but can re-enter the cell cycle after injury. In physiological conditions, the dynamic organization of the podocyte actin cytoskeleton prevents podocytes from undergoing cytokinesis and division [8,30,31]. The presence of binucleate podocytes has been reported in renal biopsies of patients with various renal diseases, including LN, suggesting the occurrence of mitotic catastrophe. Binucleate podocytes have also been observed in association with FPE [32]. 

Levels of synaptopodin, an actin-binding protein involved in stress fiber formation and actin cytoskeleton integrity [33], decrease during LN. Several regulators of synaptopodin expression have been identified as potentially involved in LN. The calcineurin pathway, which is known to contribute to actin cytoskeleton regulation, has been studied in a lupus mouse model [34,35]. Exposure to tacrolimus, a calcineurin inhibitor, is associated with improvements in proteinuria and histological lesions. This effect may result from actin cytoskeleton stabilization, due to the inhibition of synaptopodin degradation, leading to podocyte survival and the maintenance of podocyte numbers [34,35]. Moreover, Ca^2+^/calmodulin-dependent protein kinase IV (CaMK4) is upregulated in LN patients and lupus-prone models. Inhibition of CaMK4 in lupus-prone models prevent proteinuria and preserve podocyte ultrastructure through the inhibition of synaptopodin degradation [36].

Nevertheless, the podocyte signaling pathways specifically induced in proliferative lesions, but not in non-proliferative forms, of LN remain to be identified. The C-maf-inducing protein (CMIP) pathway seems to be a good candidate for involvement. CMIP expression has been reported in several glomerulopathies [37,38,39] and has recently been studied in LN patients [38]. We found that CMIP was overexpressed in non-proliferative LN (classes II and V), but it was almost undetectable in patients with class III/IV LN. The lack of CMIP expression in these proliferative forms may be secondary to NFκB activation, which is known to repress CMIP expression [40]. Indeed, NFκB is activated in several types of proliferative glomerulonephritis involving inflammatory mediators, notably in LN, and this activation aggravates glomerular lesions by promoting inflammation [41].

The NFκB pathway has also been associated with the upregulation of ubiquitin C-terminal hydrolase-L1 (UCH-L1) [42], an important regulator of the ubiquitin/proteasome system. The potential role of UCH-L1 in LN has been demonstrated in a murine model (MRL/lpr mice). UCH-L1 was found to be associated with podocyte injury and FPE through NFκB activation [43]. UCH-L1 induction was observed in podocytes during LN and was associated with podocyte dedifferentiation in vitro [42,44]. UCH-L1 expression is correlated with the internalization of podocyte-specific markers in human glomerulonephritis [44]. Interestingly, its expression seems to be related to IC-mediated glomerulonephritis, as observed in LN, and is only very weakly expressed in renal biopsy specimens from patients diagnosed with MCD, a glomerular disease characterized by the absence of kidney parenchymal inflammation [44,45], suggesting a possible link between UCH-L1 and IC-related mechanisms of injury.

## 3. Proliferative Lupus Nephritis (LN) with Crescent Formation

Crescentic glomerulonephritis (CrGN) is a severe form of kidney disease characterized by a glomerular syndrome associated with acute renal failure leading to ESRD in up to 30% of patients [46]. Histological findings are characterized by a proliferation of local glomerular cells and inflammatory cell infiltration in the Bowman’s space, forming a crescent. This proliferation leads to glomerulotubular obstruction and disconnection, causing acute kidney injury and long-term nephron loss [47]. CrGN occurs as a complication of a number of immune diseases, but a recent epidemiological study on 528 renal biopsies displaying CrGN showed that 35% corresponded to patients diagnosed with LN [48]. Crescentic LN, which is usually defined as the presence of crescents in more than 50% of glomeruli, is observed in 6–12% of LN cases [49,50]. Moreover, regardless of the proportion of glomeruli affected, crescentic lesions are found in about half of LN patients [49,50]. CrGN is a well-known risk factor of ERSD during the course of LN [48,50,51].

The pathophysiological mechanisms of crescent formation involve multiple pathways, beginning with the local release of inflammatory mediators, and ultimately leading to local epithelial cell proliferation in the Bowman’s space [52]. Podocytes have been shown to contribute to crescent formation in gene tagging experiments in a mouse model of CrGN [53] and by immunohistochemistry on human biopsy specimens [54]. During crescent formation, podocytes undergo a change in phenotype, with the loss of their specific markers [53]. Dedifferentiated podocytes can migrate [53,55] and re-enter the cell cycle [53,56]. There is evidence supporting the involvement in crescent formation of several signaling pathways, including the Janus kinase/signal transducer and activator of transcription 3 (JAK/STAT3) signaling pathway, which is activated in lupus CrGN [57] and contributes to podocyte dedifferentiation by inducing the heparin-binding EGF-like growth factor/epidermal growth factor receptor (HB-EGF/EGFR) pathway [58,59]. STAT3 can activate downstream mir-92a expression, thereby inhibiting p57 and stimulating podocyte entry into the cell cycle [60].

However, there is growing evidence to suggest that parietal epithelial cells (PECs) play a major role in crescent formation [61]. PECs were identified as the main component of crescents in a mouse model of CrGN in genetically tagged animals [62]. PECs are quiescent cells that line the Bowman’s capsule. PECs can be activated (as in CrGN), leading to the upregulation of CD44, a hyaluronic acid receptor, which facilitates their proliferation, migration, and accumulation in the Bowman’s space through induction of the Extracellular signal-regulated kinase (ERK) signaling pathway [63,64]. The mechanisms leading to CD44 induction in CrGN may involve growth factors such as Platelet Derived Growth Factor (PDGF) [61,65,66,67] and HB-EGF [59,63].

There is compelling evidence for crosstalk between podocytes and PECs in some proliferative glomerular diseases. First, the downregulation of podocyte proteins, such as podocin and synaptopodin, is correlated with PEC activation in several models of CrGN [62,68]. Furthermore, in several models of CrGN, the invalidation of specific proteins from podocytes leads to crescent formation with PEC proliferation. Previous studies have, thus, elegantly demonstrated an association between podocyte injury and HB-EGF production, leading to PEC activation through a paracrine effect [59,63]. In addition, the knockout of Krüppel-like factor 4 (KLF4), a negative regulator of STAT3, in podocytes is associated with CrGN development [69]. As suggested by in vitro findings, the mechanism underlying CrGN may be STAT3 pathway activation in podocytes, leading to “mitotic catastrophe”, triggering IL-6 release and PEC activation [69].

Finally, podocytes may maintain PECs in a quiescent state by secreting CXCL12 and inhibiting Notch in PECs [70,71]. These functional interactions highlight the crosstalk between podocytes and PEC activation. It remains unclear whether PEC activation plays also a role in podocyte regeneration [72,73].

## 4. Podocytes as Immune Cells

LN mechanisms involve multiple immune pathways in which podocytes become a target of adaptive immunity (since podocytes could express several target antigens [25,26]) and innate immunity (as podocytes expresses toll-like receptor 9 (TLR9) during LN [74]). Moreover, there is rising evidence of the direct involvement of podocytes as an immune actor in the LN process since podocytes could cross-talk with other immune cells. 

Podocytes may contribute to adaptive immunity by acting as antigen-presenting cells (APCs). Under inflammatory conditions, podocytes express major histocompatibility class II molecules [75], which contribute to lymphocyte activation, as observed in the murine model of nephrotoxic serum glomerulonephritis. The close contact between podocytes and T cells required for antigen presentation was observed in human renal biopsy species and in experimental models of LN. Moreover B7-1 (CD80), which is usually expressed on B cells and APCs and induces T-cell co-stimulation, has also been detected on podocytes in several models of proteinuric kidney diseases, including LN [76]. However, recent studies have reported conflicting results suggesting that B7-1 is not expressed in mouse models of LN and in human LN [77,78]. Moreover, clinical trials in patients with LN failed to demonstrate a clear outcome benefit of abatacept, an inhibitor of B7-1/CD28 interaction [79,80]. Further studies are required to clarify the involvement of B7-1 in LN, and in podocyte injury in particular. 

In vitro data have revealed an association between expression of CaMKIV, which is involved in podocyte homeostasis [36], and the podocyte expression of CD86, another molecule involved in T-cell activation, following exposure to IgG from patients with LN [81]. Moreover, the neonatal Fc receptor may contribute to IgG internalization in podocytes during LN because the inhibition of this receptor is associated with lower levels of IgG entry into cultured podocytes [81].

Podocytes may also contribute to the inflammatory response in LN by secreting proinflammatory cytokines [82]. Moreover, a major innate immune pathway, the NLRP3 inflammasome, may play a role in podocyte injury. Indeed, the NLRP3 inflammasome is associated with the secretion of IL1ß and IL-18 [83]. Fu et al. showed that podocytes were expressed the NLRP3 inflammasome in human LN. In an experimental model of LN, they found a close relationship between NLRP3 activation and the occurrence of proteinuria [84]. Guo et al. highlighted the potential role of necroptosis in podocytes, with their identification of RIP3 as an activator of the NLRP3 inflammasome pathway [85]. The inhibition of RIP3-dependent necroptosis in a lupus-prone model resulted in lower proteinuria and a lower severity of glomerular lesions, together with NLRP3 inflammasome inhibition [85]. These findings strongly suggest that the necroptosis pathway is involved in podocyte NLRP3 activation.

## 5. Podocyte Genetics in Lupus Nephritis

Podocyte genetics may play a major role in LN development. Two common variant alleles of apolipoprotein L1 (ApoL1) in the African-American population were recently identified as risk factors for focal segmental glomerulosclerosis (FSGS) and ESRD [86]. These variants (G1S342G and G2 insertion/deletion) have also been implicated in other renal diseases, such as HIVAN (HIV-associated nephropathy) [87], and sickle cell disease nephropathy [88]. These risk alleles were also found to be associated with the collapsing form of FSGS and with ESRD risk in a population of Afro-Americans with LN [89,90]. The mechanism underlying ApoL1-associated podocyte injury remains unclear. It has been suggested that ApoL1 variants are involved in altered vesicle trafficking, autophagosome disruption, and autophagy disturbance, leading to podocyte loss [91]. The overproduction of interferon-α, as observed in LN, may constitute a second hit, triggering podocyte injury in the presence of ApoL1 risk alleles [6,92]. Furthermore, soluble urokinase-type plasminogen activator receptor (suPAR), a molecule associated with immune system activation [93], has been shown to interact with APOL1 and αvβ3 integrin, leading to podocyte damage. The potential contribution of these interactions between podocyte molecules to the pathogenesis of LN requires further investigation. Hayek et al. have identified suPAR as a potential injury molecule in LN, based on their observation of a direct interaction between suPAR and pathogenic variants of Apol1 shown to be associated with integrin activation and podocyte detachment [94].

Podocyte-specific genetic variants, such as a homozygous variant of NPHS1 (nephrin coding gene), which encodes nephrin, have occasionally been described in patients with resistant LN [95]. Thus, podocyte-specific pathogenic genetic variants should probably be investigated in resistant LN forms, as in adult FSGS cohorts [96]. 

## 6. Lupus Podocytopathy: A Specific Form of LN

Lupus podocytopathy, a new form of LN, was recently described as an additional form not included in the usual LN classification. Lupus podocytopathy, in patients diagnosed with SLE, is characterized by the presence of MCD or FSGS lesions on renal biopsy, without subendothelial or subepithelial IC deposits. This entity was first described in case reports, and it was subsequently confirmed in case series [97,98,99,100]. 

The usual presentation of this clinical and pathological entity is very similar to that of idiopathic nephrotic syndrome (INS), with a sudden onset and massive proteinuria [100,101,102,103] (Table 2). Lupus podocytopathy seems to be particularly frequent in young women. Moreover, the onset of nephrotic syndrome in patients with lupus podocytopathy is often associated with a flare-up of lupus. Lupus podocytopathy is usually diagnosed within six months of lupus onset, and the incidence of podocytopathy appears to be higher in lupus patients (1.6%) than in the general population [100,103,104,105]. These features suggest that lupus podocytopathy is a specific entity and that there is a close pathophysiological link between SLE and podocyte injury rather than a fortuitous association.

Lupus podocytopathy is generally characterized by MCD or FSGS lesions with no subepithelial or subendothelial deposits, but mesangial deposits or minimal mesangial proliferation have been also observed [102,103,104]. Almost all patients meeting the criteria for lupus podocytopathy present more than 70–80% FPE [102,103,104]. There is no correlation between histological findings (MCD or mesangial proliferation) and clinical presentation or response to treatment [103]. Nevertheless, FSGS lesions seem to be more severe (with frequent acute kidney injury and a poorer response to immunosuppressive treatment) than MCD or mesangial proliferation in lupus podocytopathy [103]. 

Remission rates are similar in patients with class II LN and patients with lupus podocytopathy (defined as >50% FPE and nephrotic syndrome) with mesangial proliferation. However, patients with lupus podocytopathy are more likely to receive steroid monotherapy than class II LN patients, who are frequently treated with additional immunosuppressive treatment. The risk of relapse is reportedly higher for patients with lupus podocytopathy (52%) than for those with class II LN (24%) [104]. These data highlight the need to distinguish between classical class II LN and lupus podocytopathy [4].

## 7. Conclusions

**Figure 1:**
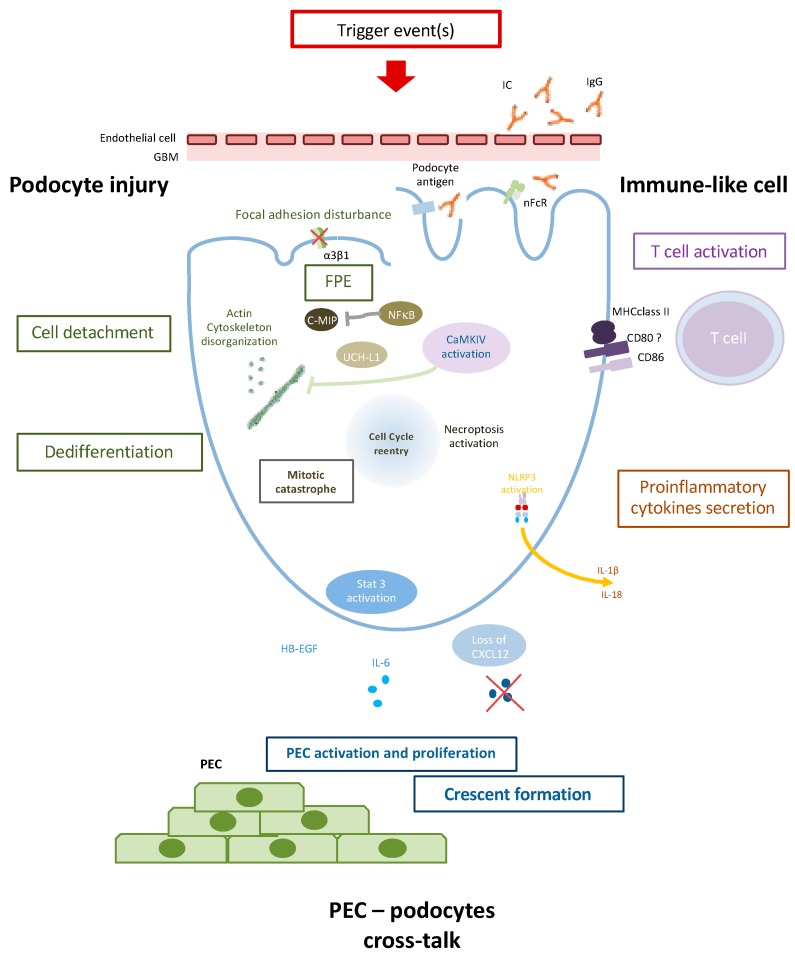
Podocyte contribution to glomerular lesion in proliferative Lupus nephritis.

Podocyte injury appears to play a key role in LN. It involves several mechanisms, including dysregulation of the podocyte actin cytoskeleton, and cross-talk with PEC and immune cells (Figure 1). Podocyte injury can lead to lupus podocytopathy, a newly characterized form of LN, but may be also involved in glomerular crescent formation. Characterization of the specific molecular and pathophysiological mechanisms involved in LN podocyte injury would facilitate the development of new therapeutic strategies for preserving glomerular integrity and renal function.

During proliferative lupus nephritis, several mechanisms could be observed:

1. Podocyte injury is characterized by FPE, loss of podocyte-specific markers, and cell detachment. Actin cytoskeleton disorganisation plays a major in FPE and cell death through mitotic catastrophe. UCH-L1 could as well contribute to podocyte injury by modulating specific podocyte protein expression.
2. Podocytes could contribute to the inflammatory process as an APC (antigen-presenting cell). Immunoglobulin (Ig) internalization through neonatal Fc receptors (nFcR) leads to CaMKIV activation and CD86 expression. CD80 and MHC may also be expressed on podocytes during inflammatory process and could contribute to T cell activation. NLRP3 inflammasome activation in the RIP3-dependent pathway could lead to proinflammatory cytokine secretion such as IL-1β and IL-18.
3. Podocyte injury finally triggers PEC (Parietal epithelial cell) activation and proliferation through, notably, the Jak/Stat pathway, HB-EGF, and IL-6 production and/or loss of CXCL12 secretion leading to PEC activation and crescent formation.

## Figures and Tables

**Table 1:** International Society of Nephrology/Renal Pathology Society (ISN/RPS) lupus classification and definition of lupus podocytopathy.

Class I	Minimal mesangial lupus nephritis	Light microscopy: normal glomeruli Immunofluorescence: possible mesangial immune deposits
Class II	Mesangial proliferative lupus nephritis	Light microscopy: mesangial hypercellularity and/or mesangial matrix immunofluorescence: mesangial immune deposits/possibly a few isolated subepithelial or subendothelial deposits
	Lupus podocytopathy *	Light microscopy: normal glomeruli/isolated mesangial hypercellularity/focal and segmental glomerulosclerosis (FSGS) Immunofluorescence: no subendothelial or subepithelial deposits, but possible mesangial deposits Electron microscopy: extensive foot process effacement (FPE)
Class III	Focal lupus nephritis	Light microscopy: endo- and/or extracapillary glomerulonephritis involving <50% of all glomeruli Immunofluorescence: subendothelial immune deposits
Class IV	Diffuse lupus nephritis	Light microscopy: endo- and/or extracapillary glomerulonephritis involving >50% of all glomeruli Immunofluorescence: subendothelial immune deposits
Class V	Membranous lupus nephritis	Light microscopy: Morphological aspects of membranous nephropathy. Immunofluorescence: subepithelial immune deposits Possible combination with class III or IV
Class VI	Advanced sclerotic lupus nephritis	≥90% of glomeruli sclerotic

* According to the definition of Bomback et al. [4].

**Table 2:** Published studies on lupus podocytopathy.

Author, Year N. pat.	Inclusion Criteria	Clinical Presentation	Histology			Prognosis	
			Light Microscopy	Ig Deposits	FPE	Response	Relapse and Hist. Trans.
**Hertig, 2002 [100] *n* = 11**	Lupus dg * NS MCD/FSGS	Female 27 years NS: 100%Hu: 30%AKI: 55% Time onset LES/NS: 5 months	Ms proliferation: 0% MCD: 30% FSGS: 70%	Ms deposit: 30%	-	CR: 70%	Relapse: 30% Hist. Trans.: -
**Kraft, 2005 [102] *n* = 18**	Lupus dg * MCD/FSGS Class I/II appearance	Female 30 years NS: 45%Hu: 60%AKI: 20% Time onset LES/NS: 1 month (in NS range)	Ms proliferation: 75% MCD: 15% FSGS: 45%	Ms deposit: 75%	FPE: >80% in 90% of NS	CR/PR: 75%	Relapse: -
**Hu, 2016 [103] *n* = 50**	Lupus dg * NS MCD/Class I/II or FSGS with FPE > 50%	Female 30 years NS: 100%Hu: 20–30%AKI: Class I/II appearance: 20%; FSGS 70% Time onset LES/NS: 5 months	Ms proliferation: 74% MCD: 26% FSGS: 18%	Ms deposit: 90%	FPE: >70% in 90% of patients	CR: 75%PR: 20%	Relapse: 50% Hist. Trans.: 40%
**Wang, 2017 [104] *n* = 31**	Lupus dg * NS Class II appearance FPE > 50%	Female 30 years NS: 100% Hu: 20%AKI: 30% Time onset LES/NS: 6 months	Ms proliferation: 100%	Ms deposit: 100%	FPE: >70% in 75% of patients	CR: 85%PR: 10%	Relapse: 50% Hist. Trans: 60%

* According to the American Rheumatologic Association criteria. N. pat. : Number of patients. MCD: minimal change disease; FSGS: focal segmental glomerulosclerosis; Ig: immunoglobulin; NS: nephrotic syndrome; Hu: hematuria; AKI: acute kidney injury; Ms: mesangial; FPE: foot process effacement; CR/PR: complete/partial remission; Hist. Trans: histological transition.
